# Considerations for Tungsten Carbide as Tooling in RFSSW

**DOI:** 10.3390/ma17153799

**Published:** 2024-08-01

**Authors:** Ruth Belnap, Taylor Smith, Arnold Wright, Yuri Hovanski

**Affiliations:** 1 Ira A Fulton College of Engineering, Department of Manufacturing Engineering, Brigham Young University, 265 CTB, Provo, UT 84602, USA; 2 Bond Technologies, 1353 Wade Dr., Elkhart, IN 46514, USA

**Keywords:** refill friction stir spot welding, RFSSW, tungsten carbide WC-Co, tool wear

## Abstract

Tool wear is a key issue for the manufacturing performance of refill friction stir spot welding in high-volume manufacturing environments. As such, the aim of this study is to examine conditions in which tungsten carbide with a cobalt binder can succeed as a tool material in the spot welding of 2029 aluminum for a sustained lifetime. Critical factors are shown herein to include cleanliness and thermal management. The life of a WC-Co toolset is demonstrated to be approximately 2998 welds, which is of the same scale as conventional steel tooling. With a WC-Co shoulder and probe, the H13 clamp showed the only significant wear.

## 1. Introduction

Friction stir welding (FSW) is a solid-state joining process that was invented in 1991 at TWI [1]. The process uses a single non-consumable tool and has been effective in producing linear and spot joints in a variety of materials. Successful welds have been performed in aluminum, steel, copper, nickel, magnesium, titanium, metal matrix composites (MMCs), polymer matrix composites, and polymers [2,3,4,5,6,7,8,9], including dissimilar combinations of many of these. The tool materials examined in research include steels, Ni-based superalloys, refractory metals, carbides, MMCs, polycrystalline cubic boron nitride (PCBN), and polycrystalline diamond (PCD) [10,11].

**Figure 1:**
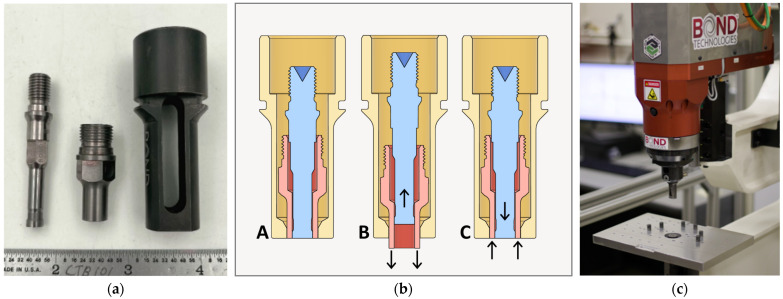
RFSSW equipment. (**a**) Example of WC RFSSW toolset used in this study; from left to right is the probe, the shoulder, and the clamp; (**b**) schematic of RFSSW process with a yellow clamp, a blue probe and a red shoulder; phase A is the clamping stage, B is the plunging stage, and C is the refilling stage; (**c**) bond RFSSW machine used in this study.

Refill friction stir spot welding (RFSSW) was patented in the U.S. in 2004 [12] with a new tool design, depicted in Figure 1a. This three-piece toolset, comprising a shoulder, a probe, and a clamp, creates a spot weld that is strong, light, and hermetic and has a flat and shiny surface finish. It accomplishes this in three phases (diagrammed in Figure 1b). First, the non-rotating clamp applies a downward force on the workpiece. Then, the rotating shoulder plunges while the rotating probe retracts, which stirs and displaces material into the chamber of the shoulder. Finally, the shoulder and probe return to zero, refilling the material and leaving a flat and flush weld.

RFSSW has been investigated in a variety of materials, comprising aluminum, magnesium, steel, copper, titanium, composites, and polymers, including dissimilar combinations [13]. Research in the last twenty years has largely investigated parameterization [14,15,16,17,18] and focused on cycle times [19,20]. While these studies have predominantly relied on steel for tooling, recent papers have revealed limitations of steels in regard to longevity.

The first limitation is the deterioration of the weld quality over a tool’s life. Montag studied this in 2014 across 3467 spot welds in 6082-T6 and detailed the degradation of the weld surface quality [21]. De Carvalho later investigated the performance of an H13 shoulder across 2500 welds in 6061-T6 and saw measurable tool wear, a consequential decline in ultimate lap shear strength (ULSS), and a change in fracture mode [22].

Uncoated steel tools are further limited in aluminum applications because intermetallic compounds (IMCs) form during welding and seize the movement of the toolset. Larsen reported that it takes approximately 70 welds for a toolset to fill up with aluminum and bind with IMCs [23]. This necessitates the disassembly of the toolset for chemical cleaning, which is impractical for most high-volume manufacturing settings. 

Specifically, 2xxx series aluminum has proven to be a challenge for RFSSW tools. Nasiri welded in 2099-T83 and seized two steel tools after fewer than 10 welds and concluded that the lithium-rich aluminum reacted with the tool material and caused metal embrittlement [24]. De Castro was able to weld in 2198-T8, also with a steel tool, for 2350 spots, but demonstrated significant wear, particularly on the outside of the shoulder. This wear was correlated with a trend of a decrease in the ultimate lap shear strength (ULSS) of 720 N for every 1000 welds [25]. This trend is contrary to the conclusion, drawn in Montag’s study of RFSSW in 6082-T6, that there is no relationship between tool wear and the ULSS [21]. 

Coatings have been shown to be effective in prolonging tool life, but, as demonstrated by Lauterbach [26], after the coating wears through and exposes the steel substrate, the same two problems (wear and IMC growth) are still present. Thus, this study is interested in establishing a non-ferrous material that may function as either a bare tool or a robust substrate. 

Tungsten carbide with a cobalt binder (WC-Co) has precedence as tooling for both linear FSW and traditional FSSW, in both steel [27,28] and aluminum matrix composites (AMCs) [29,30]. WC-Co’s hardness is greater than that of steel, which is promising from a tool wear perspective. Moreover, WC does not react with aluminum, meaning that the binding associated with IMC growth will not be a concern. It is worth noting, however, that the cobalt binder does carry the possibility of reacting with aluminum [31], and it remains to be seen whether this will be problematic in this application. Additionally, recent research has demonstrated WC-Co’s ability to produce robust and well-consolidated joints with small heat-affected zones [32]. Therefore, this paper will present findings on the overall lifetime performance of tungsten carbide as RFSSW tooling.

## 2. Materials and Methods

### 2.1. Overview

This study was performed exclusively in aluminum alloy 2029-T8 (see Table 1) in a lap configuration with a 1.52 mm top sheet and a 2.54 mm bottom sheet. This material was selected because it will wear the tool aggressively; a toolset that effectively withstands the severe conditions of 2xxx aluminum would likely last longer in a softer 5xxx or 6xxx aluminum. Bond Technologies provided both the RFSSW machine, seen in Figure 1c, and the toolsets, in Figure 1a. The probe and the shoulder were each composed of monolithic 618 WC-Co and had coarse 6-micron grains and 18 wt% cobalt binder. This grade was selected because coarser grain sizes are better from a tool wear perspective [30], and a higher percentage of binder is correlated with a tougher WC-Co [33].

This study consisted of two phases: weld development and life studies. Weld development aimed to design a weld program that generated reliably strong and well-consolidated welds, as evidenced by a sample of 30 lap shear tensile tests. Life studies consisted of a toolset performing this weld, and only this weld, until failure. 

One toolset was reserved for weld development alone. Four additional shoulders underwent four life studies, failing at 143, 144, 166, and 2998 welds in succession. Throughout the fourth life study, the performance of an uncoated clamp ring was tested against that of a coated clamp ring. The uncoated clamp was used for the first 1000 welds and the coated clamp was used for the rest of the study. Both clamps were received as identical H13 clamps, and one was subsequently coated. The coating was 4–12 microns thick and it consisted of a primary layer of titanium carbide, applied with chemical vapor deposition (CVD), and a secondary layer of molybdenum and tungsten disulfide, applied by dynamic compound deposition (DCD) [34].

The majority of the life study welds were performed on plates roughly 6″ by 12″ (although the exact size was considered arbitrary). The plates were welded in a cycle of five—meaning that any given plate would receive every fifth weld—to allow time to cool down between welds, and they were used until they were completely filled with spot welds. 

### 2.2. Weld Details 

As described earlier, an RFSSW is split into three phases, but there was an additional optional stage included in each weld of this study. The phases are clamping, plunging, refilling, and cleaning. 

During the clamping stage, the toolset approaches the workpiece at 1000 mm/min, stepping down to 350 mm/min and then to 50mm/min as it moves closer to the sheet. In the plunge, detailed in Table 2, the shoulder is programmed to descend 2.778 mm into the 4.0 mm stack-up over 1.5 s, and the refill subsequently finishes the weld in 0.5 s. Further discussion of the details of this specific weld program can be found in previous research [32].

Note that the shoulder retracts by only 2.078 mm, meaning that it finishes the weld 0.7 mm lower than it started. This difference between the shoulder’s starting position and its ending position is referred to as the tamp, and it is intended to ensure consolidation and flatness. The tamp is tuned to compensate for the material lost to flash during welding, as well as to counter the deflection of the C-frame on which the machine is mounted. These two factors (material loss and deflection) mean that the actual tamp depth will differ from the commanded tamp depth. 

The cleaning stage consists of the probe cycling up and down within the shoulder while rotating. This repeated stroke happens up to ten times and enables the buildup of aluminum between the shoulder and the probe to shear off and evacuate the toolset. As the aluminum accumulates, the forces on the toolset increase. As it cleans out, the forces decrease. 

In the present study, the cleaning stage consisted of at least one cycle after every single weld, but also in between welds according to the judgment of the welders. The isolated cleaning cycles were employed to clean out the tool when the forces were particularly high and happened as many times as necessary to decrease the forces back to a safe level. 

### 2.3. Toolset Evaluations 

The toolsets were disassembled, cleaned, and evaluated at regular intervals. Each evaluation consisted of three parts: measurements on a Starrett HB400 optical comparator (Laguna Hills, CA, USA), pictures on the Keyence VHX-7100 microscope (Mechelen, Belgium), and weigh-ins using a Mettler Toledo ME104E analytical balance (Greifensee, Switzerland). 

**Figure 2:**
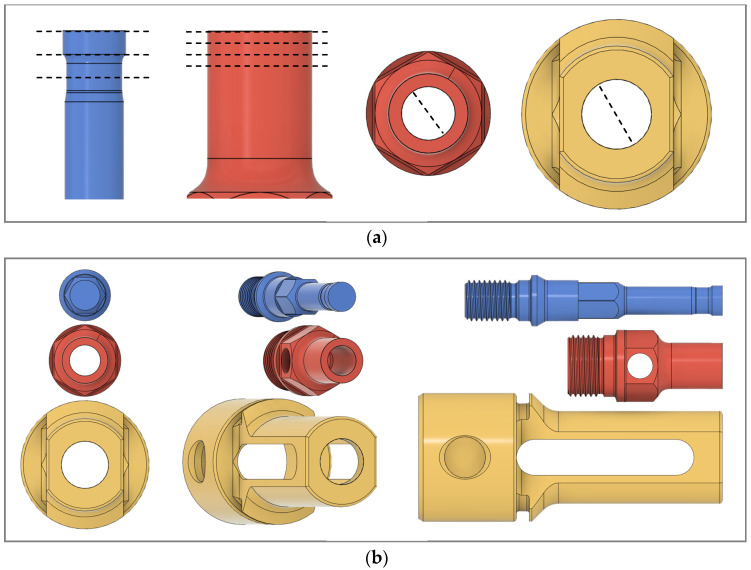
Illustrations of tool evaluations. (**a**) Models of the tools with dotted lines indicating where they were measured at each evaluation; (**b**) models of the tools illustrating the angles at which the tools were photographed for each evaluation; from left to right is 0°, 20°, and 90°.

The measurements on the shoulder included the inner diameter and the outer diameter at four points along the shaft: 0 mm, 1 mm, 2 mm, and 3 mm from the face, as diagramed in Figure 2a. The probe’s outer diameter was measured at three points along its shaft, at 0 mm, 2 mm, and 4 mm from the face, and the clamp’s inner diameter was recorded.

On the Keyence, photos were taken from three perspectives: the side, the face, and the face at a 20° angle, as shown in Figure 2b. Finally, the weights of each of the three pieces were recorded to 0.1 mg precision. Each measurement—for both the weight and diameter—was taken three times to counteract gage variation and the average was recorded.

### 2.4. Joint Evaluations

**Figure 3:**
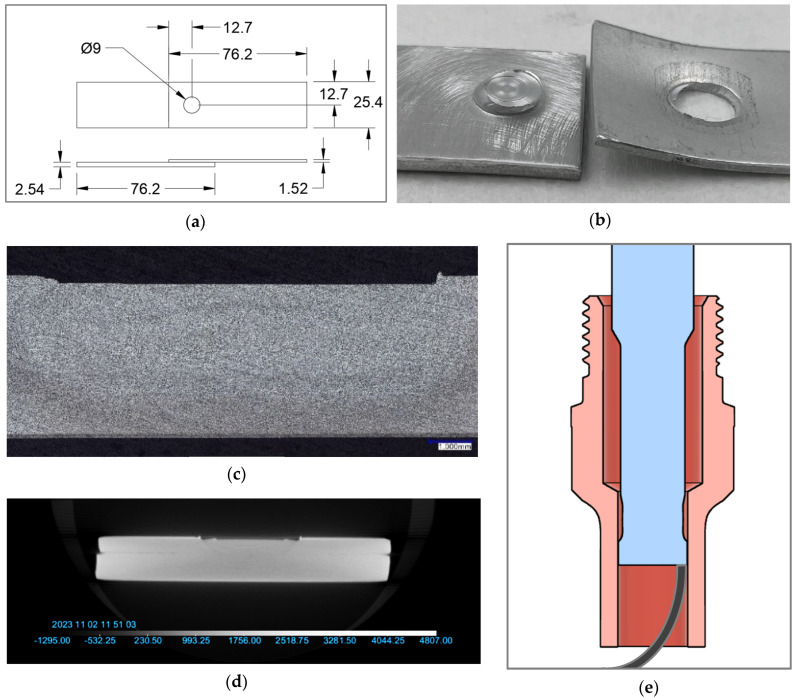
Depictions of various testing details. (**a**) Drawing of single-spot coupons for quasi-static tensile testing in shear; (**b**) example of nugget pull-out failure mode; (**c**) example of macrograph of weld cross-section; (**d**) example CT scan of weld sample; (**e**) diagram depicting placement of thermocouple during coolant tests.

Throughout each life study, the 6th to the 10th of every hundred welds were welded onto single-spot coupons and reserved for testing. These coupons were each 76.2 mm by 25.4 mm and were welded on a 25.4 mm square overlap, as shown in Figure 3a. In preparation for welding, the faying surfaces of each coupon were cleaned with isopropyl alcohol and then sanded with 80-grit sandpaper on an air-powered die grinder; this preparation happened no more than 24 h before welding. 

From each set of five coupons, three were pulled on an Instron model 4204 tensile tester at least 100 h after welding. The quasi-static tensile test yields two data points that indicate the weld strength: the ULSS and fracture mode. The desired fracture mode is nugget pullout, as shown in Figure 3b, because it demonstrates that the stirred nugget is stronger than the heat-affected zone (HAZ) circumferential to the weld.

The two remaining coupons underwent other analyses, including microscopy and computed tomography (CT) scanning, examples of which can be seen in Figure 3c,d. Optical microscopy was utilized to assess polished cross-sections of the welds to validate their full consolidation and verify a flat surface finish. The CT scanner also verified weld consolidation in three dimensions, rather than just the single plane in a macro. 

### 2.5. Other Testing

This study also included thermogravimetric analysis (TGA) using Netzsch’s simultaneous thermal analyzer (Selb, Germany), the 449 F5 Jupiter. Three tests were conducted wherein a piece of WC-Co (of the same grade as the tooling) was heated to a given temperature while recording its mass every 30 s at a balance resolution of 0.1 μg. One piece was heated to 150 °C, one to 300 °C, and one to 500 °C. The pieces were held at this temperature for 60 min and subsequently returned to room temperature. Testing occurred in an inert atmosphere of pure argon. 

In addition to testing the effect of the temperature on WC-Co, further testing clarified the most effective method to cool the toolset during welding. The first method involved chilled water running continuously through the tool head (approximately 150 mm from the working end of the toolset). The second involved a 2 s blast of room-temperature compressed air aimed directly at the working end of the tool after each weld, and the third involved the same blast at a duration of 10 s. To measure the effect, a k-type thermocouple was inserted into the shoulder with the probe retracted, as shown in Figure 3e, for the 60 s immediately following each weld. 

After one round of coolant testing, another round was conducted, with one change. The interval was changed from measuring after every weld to measuring after every 6th weld. These two rounds are notated in graphics as single vs. batched tests. The batched tests are more representative of real-life welding conditions because they allow less time for cooldown between welds. 

There was also reason to test the effect of sodium hydroxide, NaOH, on WC-Co. For this, the Mettler Toledo ME104E analytical balance was again utilized to record the mass to 0.1 mg. A piece of WC-Co was soaked in an aqueous solution of 30 mL of NaOH to 70 mL of water and weighed after 0 h, 2 h, and 60 h of soaking. At weighing, it was briefly removed from the solution, rinsed in water, and dried in a vacuum chamber for a minimum of 5 min. This test was repeated in its entirety for six pieces of WC-Co.

Additionally, further microscopy was performed using the US manufacturer Thermo Fisher Scientific’s Apreo 2 scanning electron microscope (SEM) (Waltham, MA, USA). Three samples of WC-Co were imaged: one was an untreated control sample, one had spent at least 2 h in a solution of NaOH, and the last had been heated to 500 °C for an hour, in addition to receiving the same NaOH treatment. 

## 3. Results and Discussion

### 3.1. Preliminary Development 

The weld program yielded welds that were well consolidated and flat, verified by cross-sections and microscopy. Tensile testing this weld in the lap shear configuration yielded an average ULSS of 10,239 N with a standard deviation of 1160 N; this result exceeds the standard of 3635 N for a RSW in 1.6 mm aluminum [35] by a factor of 2.8, and thus it was deemed suitable for the purposes of this study. 

**Figure 4:**
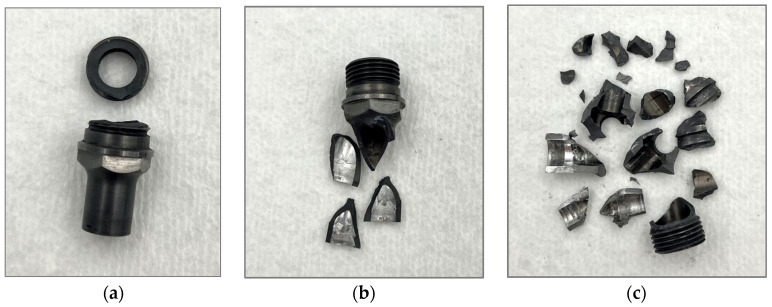
WC shoulder failures. (**a**,**b**) Examples of WC tools that failed during the cleaning cycles. (**c**) Example of a WC tool that failed during a weld.

**Figure 5:**
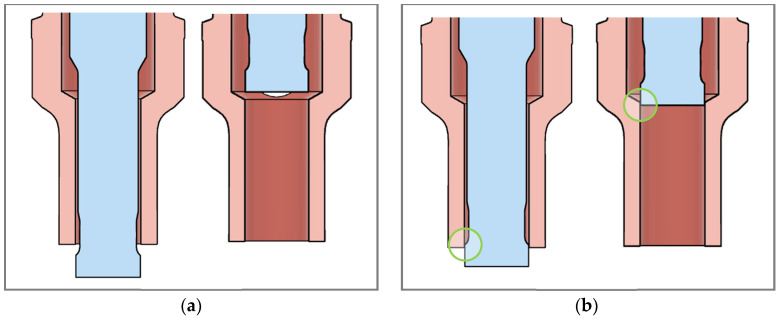
Illustrations of the movement of the probe during the cleaning stage. (**a**) Example of upper and lower points of the cleaning stroke used in early testing; (**b**) example of cleaning stroke used for later testing, with circles highlighting that the probe’s tip does not completely clear the shoulder chamber.

Initial attempts to sustain a toolset for a significant length of time included several failures. The first two shoulders of this study fractured during the cleaning cycle after the 146th and 143rd weld, respectively, imaged in Figure 4a,b. Addressing this issue involved editing the stroke of the probe during the cleaning stage from one that completely cleared the inner chamber of the shoulder, as shown in Figure 5a, to one that retained the tip of the probe within the shoulder chamber, as indicated in Figure 5b. This ensured that the probe was not able to crash into the shoulder at the top or the bottom of its stroke.

After changing the cleaning stroke, a third shoulder shattered during its 166th weld, shown in Figure 4c. This failure was different from the previous in timing and in fracture mode: where prior shoulders cracked into a few pieces during the cleaning stage, this shoulder shattered completely during the refill stage of the weld. Furthermore, it occurred within the first 10 welds of this day’s welding session, whereas the others had occurred after 40–50 welds. These differences suggest that although the cleaning stage did affect the previous fractures, there were further issues to address in order to achieve a long life.

Two hypotheses emerged at this time, one centered around thermal factors and the other around chemical factors. First, the temperatures inside a single RFSSW can approach 600 °C [32], and WC-Co is known to have a temperature limitation starting at this range [36]. So, it was reasoned that the WC-Co tooling was weakened over time at the studied temperature. As such, testing began to clarify the effect of these temperatures on the WC-Co tooling and develop effective cooling methods.

Additionally, a separate but concurrent project within the research center [37] utilized a WC-Co toolset to perform 1000 welds in 2xxx aluminum with no substantial wear on the shoulder. Critically, this project never required the toolset to be dismounted and cleaned, which generated the hypothesis that the cleaning procedure itself posed an issue. This proposition was further supported by the fact that the life study tools had all failed within a hundred welds after the first cleaning. Furthermore, NaOH is known to be used in the process of breaking down WC-Co to recycle it [38,39], meaning that WC-Co is vulnerable to chemical reaction with NaOH. All of these findings together suggested strongly that there was an issue with the use of NaOH to clean the WC-Co toolsets.

At this point, the life studies halted until this matter could be clarified with further research into the thermal and chemical effects of this process on WC-Co.

### 3.2. WC-Co Investigation

#### 3.2.1. Thermal Considerations

**Figure 6:**
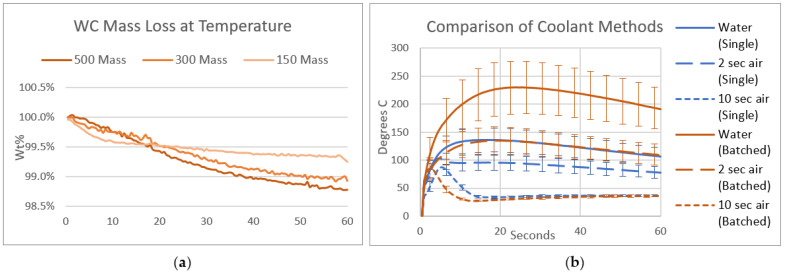
Results of two temperature tests. (**a**) Raw data of mass loss of WC over an hour at 500 °C, 300 °C, and 150 °C obtained with thermogravimetric analysis. (**b**) Average temperature (based on 4–6 replicates) of a WC toolset after welding using various coolants.

Initial tests were conducted to determine if high temperatures alone contributed to the weakening of WC-Co (indicated by a loss of mass). Figure 6a shows the results of the TGA tests and illustrates a mass loss of roughly 1.2% per hour at a given temperature, with the effect more pronounced at higher temperatures. This is interpreted to mean that parts of the WC-Co decompose at these temperatures; further investigation of this phenomenon was beyond the scope of this study. The relevant conclusion from this test was that keeping the tool below 150 °C may support long-term reliability.

Given this target, the coolant tests helped to determine the best way to keep the tool temperature low. There are two noteworthy details to discuss from the results, given in Figure 6b. The first is the similarity between the single-weld round with a water coolant and the batched-weld round with a 2 s air blast; the fact that the two curves are almost identical reveals that waiting one minute between welds provides the same cooling that a 2 s air blast does.

The second point is regarding the differences between the single-weld round and the batched-weld round. For the first two coolants (water and 2 s air), the batched-weld round maintains a higher temperature throughout the 60 s. The 10 s air blast, however, produces very similar curves for both rounds. Furthermore, the average standard deviation of the batched water test is 84, whereas that of the batched 10 s air test is only 6. Both of these findings suggest that the longer air blast consistently quenches the tool to below 100 °C, even when running welds in rapid succession.

#### 3.2.2. Chemical Cleaning Considerations

Prior to each toolset evaluation, the toolset was soaked in an aqueous solution of NaOH, which was precedented by similar studies with steel tooling [21,22,23,25]. One study did avoid the use of NaOH only for the sake of imitating a manufacturing environment; in other words, NaOH was undesirable for logistical reasons and not because its use would cause tool failure [40]. Furthermore, Liu used NaOH to clean WC-Co tooling in the linear FSW of aluminum matrix composites and did not report any negative impact [29]. Nonetheless, further investigation was warranted by the continued failures in the life studies, along with the results of the adjacent project mentioned in Section 3.1 [37].

**Figure 7:**
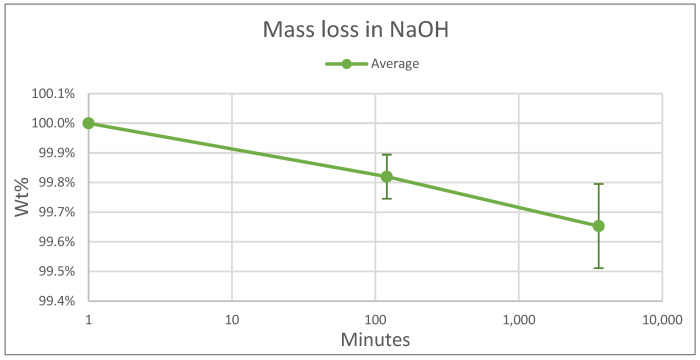
Mass loss of WC over time in a solution of NaOH. This graph depicts an average of 8 samples of WC.

The results of this paper’s investigation are depicted in Figure 7. Although the effect varied in magnitude, a mass loss was recorded over the course of the test. This outcome indicated that the WC-Co reacted with the NaOH and potentially compromised the structural integrity of the WC-Co. Also noteworthy is the fact that the initial drop over the first two hours was comparable in scale to the second drop over the next two days, suggesting that the reaction slowed down over time.

**Figure 8:**
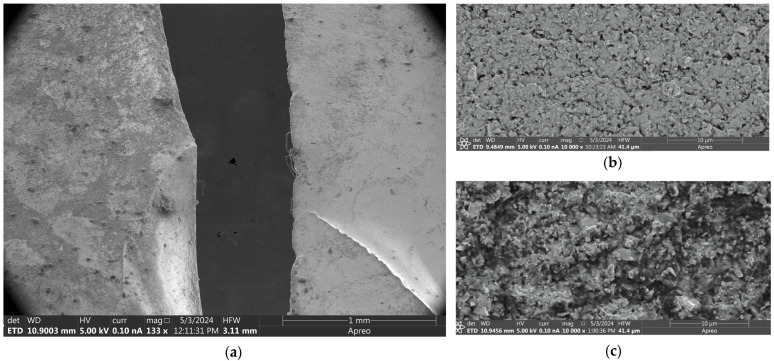
SEM images of WC. (**a**) SEM macrograph of WC treated with NaOH (on the left) and untreated WC (on the right). (**b**) SEM micrograph at 10,000× of untreated WC sample. (**c**) SEM micrograph at 10,000× of WC treated with NaOH and heat.

The SEM images further strengthen the hypothesis that NaOH reacts with WC-Co. Figure 8a shows a comparison of an untreated WC-Co sample (on the right) and a sample that has been exposed to NaOH (on the left), and Figure 8b,c depict similar samples at 10,000× *g* magnification. The untreated sample is smooth, but the treated sample is textured and dips in and out of the depth of field of the SEM, despite using identical settings. It is clear from these images that the NaOH corrodes the surface of the WC-Co, resulting in the observed texture.

The exact reaction happening between WC-Co and NaOH is documented elsewhere [41,42] and is not of specific interest in the present study. It is enough to conclude that it exists and has detrimental effects on the tool life.

Given the results of the WC-Co investigation, the researchers concluded that both chemical and thermal factors contributed to the previous tool failures in this study. Moving forward, adjustments were made to eliminate these complications in the final life study.

### 3.3. Life Study

Two significant differences existed between the fourth life study and its predecessors. The first was the cleaning method for the probe and the shoulder, which changed from chemical cleaning with NaOH to mechanical cleaning with files and wire brushes (although the chemical cleaning remained for the clamp, which was not WC-Co). The second adjustment was the cooling method, which changed to include a continuous flow of air in addition to the chilled water that had been previously utilized. With these adjustments, the tool was able to weld to 2998 welds before finally seizing and fracturing soon thereafter. Fracture occurred after dismounting the tool while attempting to unseize the shoulder from the probe.

Note that because survival to 1000 welds had already been verified by the project mentioned in Section 3.1, the first thousand welds in the final life study were performed without electively stopping for evaluations. After the first thousand, evaluations were completed every 250 welds.

#### 3.3.1. Seizures

Over the course of nearly 3000 spot welds, there were four occasions on which the toolset seized, meaning that the probe became unable to move more than ~1 mm without hitting the force limits of the machine. The seizures happened at welds 258, 338, 1007, and 2998. The gap between the third and the fourth seizure can be explained by the fact that, after the third seizure, the tool was regularly dismounted and cleaned for evaluation, which alleviated the forces on the toolset. After each seizure, the probe and shoulder were dismounted in their seized positions and then separated using a manual press. This method was effective for the first three seizures but caused the ultimate fracture of the shoulder after the fourth seizure.

The cause of the seizures is related to the accumulation of aluminum between the shoulder and the probe. During welding, aluminum builds up at two interfaces: between the shoulder and clamp and between the shoulder and probe. The aluminum that accumulates between the shoulder and the clamp has not yet proven to be problematic, as it can escape the toolset through the slots in the clamp. The material between the shoulder and the probe, however, continues to build and affects the weld forces, even despite attempts to clear it out with cleaning cycles. In other words, during welding, aluminum extrudes into the gap between the shoulder and the probe (nominally 0.1 mm), and it accumulates there until the forces required to move the probe reach the limits of the machine. It is currently unknown whether this phenomenon is caused by the aluminum reacting with the cobalt binder and forming IMCs or simply by the friction force growing in the shoulder/probe interface as the aluminum accumulates. Such determination is left to future research.

**Figure 9:**
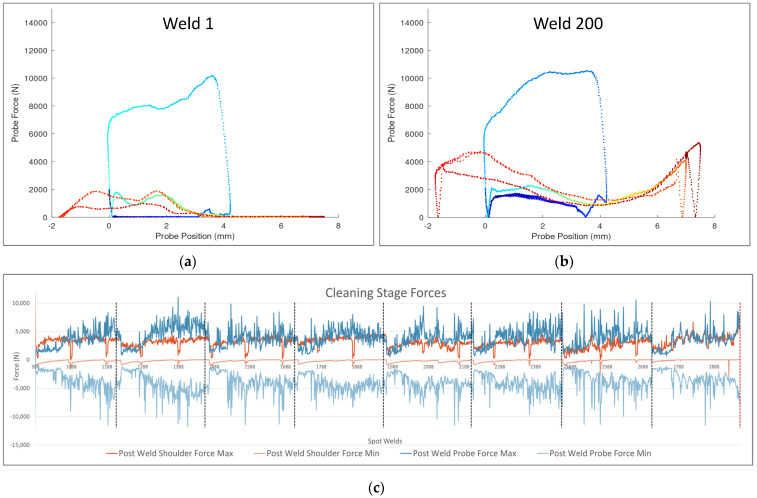
Graphs showing forces during and after welds throughout the life study. (**a**) Graph depicting probe position and force over time during the first weld of the life study, with the weld starting at dark blue and proceeding through the rainbow to dark red. (**b**) The same graph for the 200th weld of the life study. (**c**) Graph depicting the maximum forces (on top) and minimum forces (on bottom) of the probe (in blue) and shoulder (in red) during the cleaning stage following the welds in the study.

To illustrate this phenomenon, Figure 9a,b depict the relationship between the probe force and probe position over the length of a single weld. The weld begins in dark blue and proceeds through the colors of the rainbow to dark red at the end of the cleaning cycle. With a clean tool, as in Weld #1, the forces are low before the weld (in dark blue) and after the weld (in red). However, by Weld #200, the accumulated aluminum causes increased forces before, during, and after the weld, throughout the length of the probe’s stroke. As the welding continues, the probe experiences higher and higher forces until seizure.

This phenomenon can also be observed in Figure 9c, which shows the maximum tensile and compressive forces during the cleaning cycle of the probe and the shoulder throughout the final 2000 welds. The tool was demounted and cleaned for evaluation every 250 welds, which is indicated in the graph by black dotted lines. Each cleaning correlates with a decrease in the magnitude of the forces. This clearly shows that a clean tool demonstrates lower forces, which promotes smoother operation.

#### 3.3.2. Wear

The probe in this study saw little to no wear, as has been similarly documented in prior research [22,25]. However, unlike in the recorded literature, the wear on the shoulder was also minimal. Montag recorded a decrease of approximately 0.3 mm in the outer diameter of the shoulder over 3500 welds in 6082-T6 [21], and De Carvalho reported a loss of approximately 0.14 g in an H13 shoulder across 2500 welds in 6061-T6 [22]. In contrast, this WC-Co shoulder lost only 0.0008 mm in diameter and 0.0175 g over the course of 2750 welds in 2029-T8. This suggests that, with proper conditions, WC-Co tooling is likely to last significantly longer than steel tooling in RFSSW.

**Figure 10:**
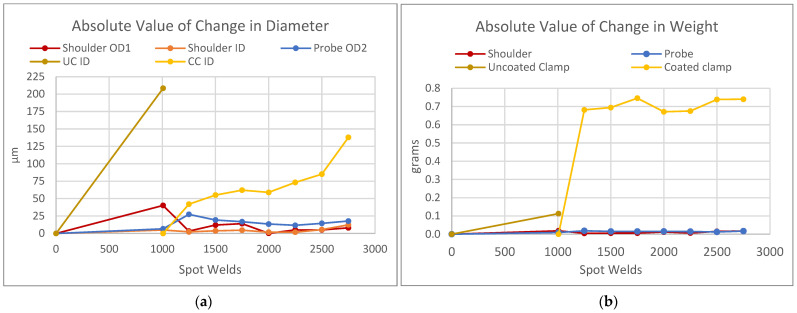
Recorded wear on tools during the life study. Each point is an average of three measurements of the given sample, and the standard deviation of these is <0.1% in all cases. (**a**) Absolute change in the diameter of the tools, with OD1 meaning the outer diameter 1 mm from the face of the tool, OD2 meaning the outer diameter 2 mm from the face of the tool, ID meaning the inner diameter at the face of the tool, UC meaning the uncoated clamp, and CC meaning the coated clamp. (**b**) Absolute change in the weight of the tools.

Unlike the probe and the shoulder, and in contrast to studies with H13 shoulders that reported no meaningful wear on the clamp [22], both clamps in this study exhibited significant wear, although they did so at different rates. The inner diameter of the uncoated clamp increased by 0.21 mm across 1000 welds and that of the coated clamp increased by 0.14 mm across 1750 welds, illustrated in Figure 10a. In other words, the coating caused a reduction in wear of 38% compared to the uncoated clamp in this study, as measured in terms of the diameter.

Measuring by weight, as depicted in Figure 10b, reveals another insight about the coated clamp. While the uncoated clamp only lost 0.11 g after 1000 welds, the coated clamp experienced a greater drop of 0.68 g after only 250 welds. After this initial drop, however, the rate of change slowed, and the coated clamp only shed 0.059 g across the next 1250 welds. This finding may indicate that the top layer of coating was removed after the first 250 welds, either because it wore off or because the NaOH cleaning solution reacted with the (W,Mo)S_2_ and removed it; further research would be necessary to confirm which of these, if either, is the case. In any case, the coated clamp subsequently proved to be wear-resistant, indicated by the comparison to the uncoated clamp.

#### 3.3.3. Weld Quality

The weld quality will be described by the strength and surface finish, with the strength measured by the ULSS and the surface finish measured by the tamp depth. The measurements in both categories deteriorated over time; the causes can be connected to clamp wear and uncontrolled boundary conditions.

**Figure 11:**
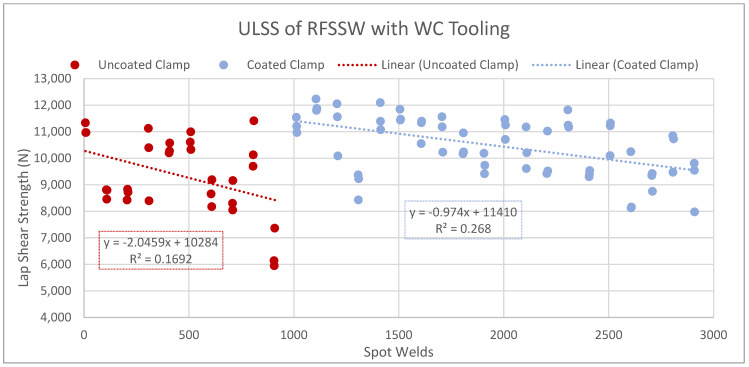
ULSS of spots across WC tool life; the first thousand welds, created with the uncoated clamp, are in red and the following welds, created with the coated clamp, are in blue.

First, the connection between the ULSS and clamp wear is illustrated in Figure 11, which shows how the ULSS changed when the uncoated clamp was changed to the coated clamp. The ULSS for the uncoated clamp follows a trend of approximately −2046 N with every 1000 welds. The change to the coated clamp is aligned with an initial increase in ULSS, but it too decreases over time, at a rate of approximately −975 N with every 1000 welds—about half of that of the uncoated clamp. These trends match the material loss of the clamps themselves (described in Section 3.3.2), suggesting that mitigating the wear in the clamps would also lessen the reduction in the ULSS.

**Figure 12:**
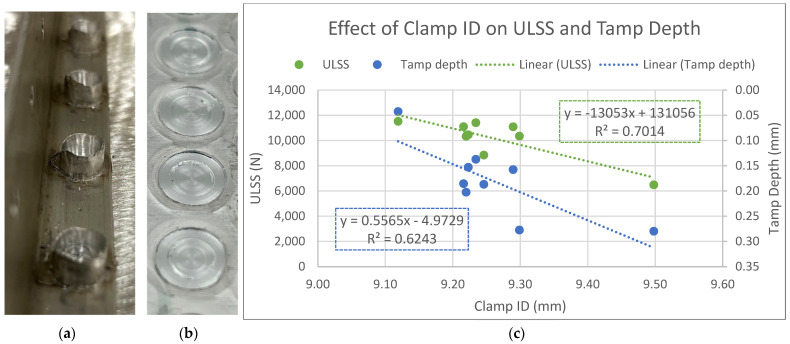
Illustrations of the results of clamp wear. (**a**) Examples of welds with extreme flashing created with a worn clamp in AA2xxx. (**b**) Examples of welds created with new clamp in the life study. (**c**) Graph showing relationship between tamp depth, ULSS, and clamp ID.

Wear on the clamp causes a decrease in the ULSS due to the increasing loss of material to flash. Figure 12a,b show examples of welds with extreme flashing after clamp wear, as well as examples of welds created with a new clamp (none of which had any post-processing). It is clear that a worn clamp allows for more material to extrude into the toolset. This phenomenon is central to the decline in weld quality over the life of this toolset.

**Figure 13:**
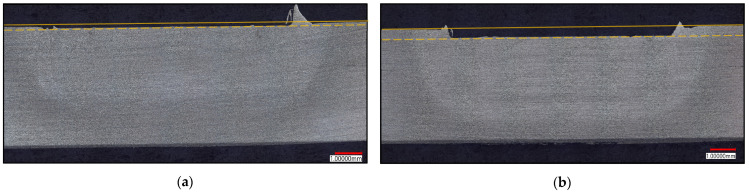
Illustration of tamp depth. (**a**) Weld 306. (**b**) Weld 808. The solid yellow line indicates the top of the base material, and the dashed yellow line represents the top of the weld. The difference between the two is the tamp depth.

A larger inner diameter of the clamp is correlated with a deeper tamp depth. The tamp depth is measured from the top of the base material to the top of the weld face; Figure 13a,b illustrate this measurement on two welds from the life study. Although the commanded tamp depth is the same for every weld, the actual tamp depth varies. This can be explained by the deflection of the large C-frame on which the machine is mounted. The position of the shoulder is controlled, but the position of the anvil deflects at a rate of approximately 0.0762 mm per 1 kN. However, as the clamp wears, more material extrudes into the toolset and relieves the pressure on the toolset, meaning that the anvil deflects less. In other words, as the clamp allows for more flash, it enables the machine to better engage with the material as commanded, leaving a lower tamp and a thinner top sheet.

**Figure 14:**
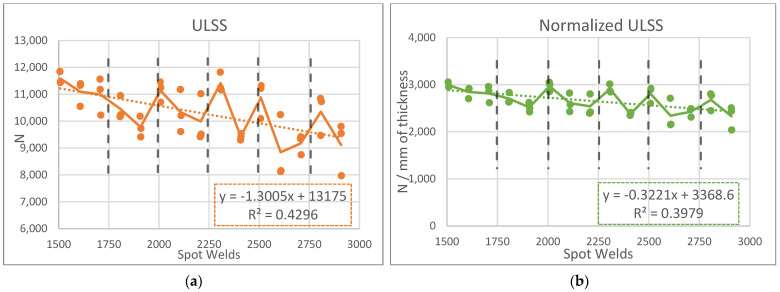
Graphs of ULSS throughout the latter half of the life study. (**a**) Graph depicting ULSS over the latter half of the life study with dashed lines indicating when the toolset was dismounted and cleaned. (**b**) The same graph with the ULSS normalized by the effective thickness to compensate for variations in tamp depth.

This is the reason that the tamp depth is correlated with both an increase in the clamp ID and a decrease in ULSS, as depicted in Figure 12c. A larger clamp ID reduces the effective thickness of the top sheet, weakening the weld. Note that the relationship between the tamp depth and ULSS has been observed before [32]. Figure 14a,b show the ULSS results with and without accounting for the tamp depth effect. Initially, the slope indicates a loss of 1300 N per 1000 welds, but, after normalizing, this slope decreases to approximately 322 N per 1000. This rate of decline is less than half of the 720 N per 1000 that De Castro reported with a steel toolset [25]. Therefore, given control of the tamp depth, the effects of WC-Co wear on the weld quality are minimal.

Furthermore, looking closer at Figure 14 reveals another insight. In addition to the overall downward trend in the ULSS, there is also a negative slope between each cleaning. This does not necessarily indicate that a clean tool produces stronger welds than a dirty tool; rather, it might suggest that colder conditions produce stronger welds than hotter conditions. Although the toolset is effectively cooled, as discussed in Section 3.2.1, the thermal inputs into the weld itself still increase during welding because the boundary conditions are not controlled. The diamond anvil heats up during welding and transfers its stored heat to the workpieces. Increased heat input in the FSW of over-aged precipitation-strengthened aluminum alloys, like 2029-T8, has a detrimental effect on the strength of the joint [43,44]. Therefore, controlling the heat input through boundary conditions is crucial to maintaining the joint quality over time.

## 4. Conclusions

The purpose of this study was to collect data on the wear characteristics of a WC-Co RFSSW shoulder and probe in a high-volume setting. A total of 2998 welds were created with such a toolset in AA2029-T8. The use of NaOH was shown to have deleterious effects on the life of the WC-Co toolset. Moreover, maintaining a lower tool temperature was demonstrated to support a longer life for WC-Co tooling. Among the three tools, the H13 clamp demonstrated the only significant wear, and this wear contributed to the deterioration of the weld quality over the life of the toolset. In a setting where clamp wear is controlled, the decline in weld quality is shown to be limited.

The key findings from this study can be summarized as follows.

- For successful long-term use as RFSSW tooling, WC-Co must be cooled to temperatures beneath 150 °C.
- For successful long-term use as RFSSW tooling, WC-Co must not be cleaned with NaOH.
- Given control of the temperature and cleanliness, WC-Co tools can last for approximately 2998 welds without presenting significant wear on the shoulder or the probe.
- In a configuration with a WC-Co probe/shoulder combination and an H13 clamp, the clamp will wear the fastest.
- Clamps coated with TiC exhibit wear approximately 38% slower than bare H13 clamps.
- Clamp wear contributes significantly to material loss during welding and a commensurate decrease in ULSS.

Future research in this area may include investigations into tool designs that inhibit aluminum buildup between the shoulder and the probe; such a design would counter the occurrence of seizures, as documented in this study. Also, the chemical makeup of the aluminum buildup between the shoulder and the probe would be of interest to discern whether Al-Co intermetallic growth is involved in this problem. Additionally, managing the effects of clamp wear will be crucial to maintaining the weld quality over the life of a WC-Co toolset. As such, alternative clamp materials, coatings, or designs that provide more wear resistance than those presented herein will be of future interest.

## Figures and Tables

**Table 1:** Chemical composition of AA2029 [32].

Cu	Mg	Ag	Mn	Zr	Si	Fe	Ti	Al
3.2–4.0%	0.8–1.1%	0.3–0.5%	0.2–0.4%	0.08–0.15%	0.12% max	0.15% max	0.15% max	Balance

**Table 2:** Weld program for WC-Co toolset in AA2029.

RPM	Step Time	Shoulder Step	Probe Step
3400	1.000	−1.778	3.229
3400	0.500	−1.000	1.000
2800	0.500	2.078	−4.279

## Data Availability

Data are contained within the article.
